# Identifying the oral microbiome of adolescents with and without dental fluorosis based on full-length 16S rRNA gene sequencing

**DOI:** 10.3389/fmicb.2024.1296753

**Published:** 2024-02-06

**Authors:** Shanshan Luo, Ruirui Shao, Yue Hong, Ting Zhang, Qingshuai Zhou, Qian Zhou, Fengqing Rao, Xingxing Zhao, Yangting Dong, Ruiyu Zhu, Ping Ling, Guzhen Cui, Zhizhong Guan, Peng Luo, Yan He, Xiaolan Qi, Jian Liao, Wei Hong

**Affiliations:** ^1^Key Laboratory of Endemic and Ethnic Diseases, Ministry of Education and School/Hospital of Stomatology Guizhou Medical University, Guiyang, Guizhou, China; ^2^He Guantun Town Health Center in Qixingguan District, Bijie, Guizhou, China; ^3^Collaborative Innovation Center for Preventionand Control of Endemic and Ethnic Regional Diseases Co-constructed by the Province and Ministry, Guiyang, Guizhou, China; ^4^Guizhou Provincial People’s Hospital, Guiyang, Guizhou, China; ^5^School of Biological and Chemical Engineering, Zhejiang University of Science and Technology, Hangzhou, China; ^6^Pediatric Intensive Care Unit, Guiyang Maternal and Child Health Care Hospital, Guiyang, Guizhou, China; ^7^Key Laboratory of Microbiology and Parasitology of Education Department of Guizhou, Guizhou Medical University, Guiyang, Guizhou, China

**Keywords:** dental fluorosis, oral bacterial, coal-burn-pollution-associated fluorosis, full length 16S rDNA sequencing, microbiome

## Abstract

Dental fluorosis, resulting from long-term environmental exposure to fluoride, is prevalent among diverse populations worldwide. Severe fluorosis not only compromises the aesthetic appeal of teeth but also impairs their functionality. This study aims to investigate the oral microbiome in dental fluorosis and the health individuals of adolescents living in the endemic fluorosis area of Guizhou, China through full-length 16S rDNA sequencing. Fourty-six individuals meet the sampling criteria, and we divided these samples into the following groups: a healthy group (*H* = 23) and a dental fluorosis group (*F* = 23), and two subgroups of Miao ethnicity: a healthy Miao group (Hm = 13) and a dental fluorosis Miao group (Fm = 15). A total of 660,389 high-quality sequences were obtained, and 12,007 Amplicon Sequence Variants (ASVs) were identified, revealing significant variations in oral microbiome between Fm and Hm groups. The composition of oral microbiota was similar between the H and F groups. At the genus level, *Pseudopropionibacterium* and at the species level, *Streptococcus oralis_subsp.dentisani_clade_058* were less abundant in group F than in group H (*P* < 0.05). Further analysis revealed that the abundance of *Capnocytophaga gingivalis* and *Kingella denitrificans* was significantly lower in Fm fluorosis patients than in the Hm group (*P* < 0.05). Based on the LEfSe analysis, the potential core biomarkers in the oral of Fm fluorosis patients were identified at different taxonomic levels, ranging from phylum to species. These include *Gammaproteobacteria*, *Prevotella sp_HMT_304*, *Gemella sanguinis*, and *Gracilibacteria_*(*GN02*). Network analysis revealed that the microbiota in the fluorosis group exhibited more complex interactions with each other than the healthy group. Notably, within the Hm group, the potential biomarkers *Capnocytophaga gingivalis* and *Kingella denitrificans* exhibited a positive correlation. Finally, we employed PICRUSt2 analysis to explore the abundance clustering of the top 30 functional units in each sample, and we found that the metabolic pathway compositions of the four groups were similar. In summary, our findings suggest that the microbial composition of plaque in Hm patients with dental fluorosis is significantly altered, and we identified the potential marker microorganisms that contribute to these changes.

## 1 Introduction

Dental fluorosis, or enamel fluorosis, is a prominent clinical symptom in the early stages of chronic fluorosis. It is a developmental enamel disorder because of prolonged exposure to excessive fluoride concentrations while developing teeth. This decreases enamel mineral content and increases enamel porosity (Gu et al., 2020). Dental fluorosis is prevalent in areas where fluoride is widely distributed, especially in groundwater with fluoride concentrations ≥1.5 ppm (Rojanaworarit et al., 2021). It has been reported in various regions worldwide, including Australia, the Americas, Brazil, India, and China (Verma et al., 2017; Do et al., 2020; Zhang et al., 2020; Guo et al., 2021; Martignon et al., 2021). Recent studies suggest an increasing trend in dental fluorosis patients due to the geological environment and the previous addition of fluoride in drinking water to prevent dental caries (Wiener et al., 2018). In addition to drinking water fluorosis, coal-fired fluorosis is another cause of dental fluorosis, and a study reported that there are more than 16.1 million cases of coal-fired fluorosis in China (Luo et al., 2011). Guizhou Province is the area most seriously affected by coal-burned fluorosis, with 19 million people living in coal-fired endemic fluorosis areas (Jha et al., 2011). The severity of dental fluorosis is influenced by various factors such as time of exposure to fluoride, autoimmunity, nutrition, bone growth, and development (Wei et al., 2019). Clinical manifestations of dental fluorosis include white cloudy changes on the enamel surface, brown, dark brown, or even black staining, and irregular enamel defects in heavily fluoridated teeth. These manifestations affect the patient’s appearance and chewing function and may also be accompanied by skeletal fluorosis, which may harm the neurological, cardiovascular, and endocrine systems (Gu et al., 2020; Yani et al., 2021). According to the Fourth National Oral Health Epidemiological Survey in China, the prevalence of dental fluorosis in children aged 12 years was 13.4%, higher than 11.7% in the third survey (Zhou et al., 2018).

After the gastrointestinal system, the oral cavity has the second-largest microbiota in the human body. And like the gastrointestinal tract, it possesses a complex and stable microbial community structure (Escapa et al., 2018; Caselli et al., 2020). With the development of molecular technology, the diverse and unique oral microbial community, or oral microbiome, is gradually being revealed. However, when the balance and homeostasis of maintaining a healthy microbiome are disturbed, it can result in various oral diseases, such as caries, periodontal disease, peri-implantitis, and oral mucosal disease (Albertini et al., 2015; Chen et al., 2018; Tanner et al., 2018; Li et al., 2022; Ptasiewicz et al., 2022; Weng et al., 2022). Therefore, exploring and researching oral microorganisms to identify specific microorganisms related to diseases can help us understand the pathogenesis of diseases and even prevent their occurrence (Serra e Silva Filho et al., 2014; Matsha et al., 2020; Dong et al., 2021; Esberg et al., 2021; Tuominen and Rautava, 2021; Xu and Han, 2022).

In recent years, 16S rRNA gene sequencing has become a common and efficient method for studying the structural diversity of microbial communities in the gastrointestinal tract and oral cavity (Yang and Que, 2020). While previous studies have used high-throughput sequencing of the 16S rRNA V3-V4 region to study microbial community structure, recent research has shown that full-length sequencing platforms provide greater taxonomic resolution and accuracy (Johnson et al., 2019). Although high fluoride concentrations are known to cause dental fluorosis, it is unclear whether fluorosis can reshape the oral microbiome and, therefore, be involved in the progression of fluorosis. We speculate that the damage of fluoride to enamel creates an environment conducive to acid-producing gram-negative bacteria, which may change the oral microbiome and even promote the formation of dental fluorosis. Therefore, in the present study, we used 16S rRNA full-length high-throughput sequencing to analyze the dental plaque samples of patients with dental fluorosis and healthy individuals. We also explored the differences in oral microbial composition between healthy and dental fluorosis individuals in the Miao population, which is predominant among dental fluorosis patients. The aim of this study is to investigate the oral microbiome in dental fluorosis and the health individuals of adolescents living in the endemic fluorosis area of Guizhou, China.

## 2 Materials and methods

### 2.1 Collection and preparation of samples in endemic coal-burn-pollution-associated dental fluorosis area of Guizhou province

The present study was carried out following the regulations established by the Guizhou Medical University Ethics Committee [Approval Number: 2019(19)], and the guardians of all participating participants gave their informed consent. Students between the ages of 9 and 18 at Suojia Primary and Middle School, located in an area of Guizhou province with a high prevalence of dental fluorosis, provided dental plaque samples. The inclusion criteria for subjects were the absence of congenital or systemic diseases (e.g., diabetes, hepatitis, HIV), no dental caries and periodontal disease, no history of alcohol or tobacco use, and no antibiotic use within 2 months before sampling. The exclusion criteria included non-local residents, patients with congenital or systemic diseases, dental caries, periodontal disease, use of antibiotics or oral care drugs within 2 months before sampling, and inability to cooperate with sampling.

We used the statistical software G*Power (version 3.1.9.6, FranZ Faul, Universität Kiel, Germany) to estimate the sample size. The significance level was set to 0.05, and the statistical power was 80%. The theoretical sample size for each group should be 26, and a total of 52 samples, but unfortunately, due to strict exclusion criteria (2), sequencing failure (3), and parental withdrawal (1), 6 samples were excluded from the study, resulting in an actual sample size of 46. All participants got a thorough oral examination that followed the WHO-recommended Dean dental fluorosis categorization criteria to guarantee uniformity. Subjects skipped meals for at least an hour before sampling and didn’t have any dental care or treatment for 24 h. Sterile cotton swabs were employed to swab the surface of participants’ teeth plaque gently and iteratively. The cotton swab heads, containing samples from the tooth surfaces, were placed into 2 ml sterile Eppendorf tubes (with excess parts broken off). The Eppendorf tubes were stored on ice, transported to the laboratory within 8 h, and stored at −80°C for subsequent sequencing analysis.

Based on the Dean’s dental fluorosis categorization criteria and ethnic origin, the samples were divided into four groups: the health group (H, *n* = 23), the fluorosis group (F, *n* = 23, including moderate and severe dental fluorosis), the healthy Miao minority group (Hm, *n* = 13), and the fluorosis Miao minority group (Fm, *n* = 15) (Supplementary Figure 1 and Supplementary Tables 1, 2). Based on the degree of dental fluorosis, the samples were categorized as healthy or fluorosis, and the Miao minority group was added to examine any potential racial influences on oral microbiota.

### 2.2 16S rRNA gene amplicon sequencing and DNA extraction

We obtained all DNA genomic samples through the OMEGA Soil DNA Kit (M5635-02) (OMEGA Bio-Tek, Norcross, GA, USA) and were directed by the manufacturer’s recommendation. The total DNA genomic samples obtained were stored at −20°C until further processing. We generated almost full-length bacterial 16S rRNA genes by PCR amplification using 27F and 1492R as primers. Then, to purify the obtained PCR products, we further purified them using agcourt AMPure Beads (Beckman Coulter, Indianapolis, IN). The products obtained were then quantified using the PicoGreen dsDNA Assay kit (Invitrogen, Carlsbad, CA, USA) and mixed into aliquots. The final sequencing results were obtained using the PacBio Sequel platform (Shanghai Personal Biotechnology Co., Ltd., Shanghai, China), which utilizes single-molecule real-time sequencing (SMRT) technology.

PacBio cycle consensus sequencing (CCS) reads were obtained from many sub-read alignments to minimize the sequencing error rate. Unprocessed sequences were processed through the PacBio SMRT Link Gateway (v5.0.1.9585). We filtered the output file at least three times to reduce noise with a minimum prediction accuracy of 99% (minFullPass = 3, minPredictedAccuracy = 99). The generated file was then modified to eliminate sequences larger than 2000 base pairs (bp).

### 2.3 Sequencing and ASVs (amplicon sequence variants) clustering analyses

We evaluate microbiome bioinformatics using QIIME2 (version 2019.4), with a few minor tweaks done in accordance with the official courses^1^ is the URL of the webpage. The sequences underwent quality using the DADA2 plugin (Callahan et al., 2016). With data reduced to 4,588 sequences per sample, beta and alpha diversity metrics were computed using the diversity analysis plugin. Non-singleton amplicon sequence variants (ASVs) were used to build a phylogenetic tree using MAFFT alignment and FastTree2. The feature-classifier plugin’s classify-sklearn nave Bayes taxonomy classifier was used to give a taxonomy to ASVs using the HOMD Database.^2^ All fresh sequences were uploaded to the NCBI Sequence Read Archive^3^ with the accession number SRP433491.

### 2.4 Bioinformatics and statistical analyses

The sequencing data were analyzed and primarily explored using the QIIME2 and R software (v3.2.0). The ASV table in QIIME2 produced ASV-level alpha diversity indexes, including the Shannon diversity index, Chao1 richness estimate, and Faith’s PD. Rarefaction curves were produced to investigate the variation trend of sample alpha diversity with rarefy depth. Using beta diversity research and UniFrac distance measuring techniques, the structural variability of microbial communities in different samples was examined. The findings were presented using principle coordinate analysis (PCoA), and the adonis was used to analyze the differences between the two groups (Lozupone and Knight, 2005). Using QIIME2’s default settings, random forest analysis was used to separate samples from several groups. Automated hyperparameter tuning and sample prediction were carried out using nested stratified k-fold cross-validation with a threshold of 10. The parameters (significance level, *P* < 0.05, and [LDA] score > 3 were used to define phenotypes) were used in LEfSe (Linear discriminant analysis effect size) to find differentially abundant taxa across groups (Segata et al., 2011). We built a co-occurrence network based on Spearman’s rank correlation with the threshold set at 0.6 coefficients, with nodes representing genera or species and edges reflecting correlations between these genera or species. Using Cytoscape, the network was made visible. Using the KEGG^4^ databases, PICRUSt2 (Phylogenetic investigation of communities by reconstruction of unobserved states) was used to predict the roles of microorganisms (Langille et al., 2013). R packages (v3.2.0), Python (2.7), and STAMP software (v2.1.3) were used to carry out the statistical analysis. When *P* < 0.05, differences were deemed significant. When *P* < 0.01 and *P* < 0.001, differences were deemed highly significant.

## 3 Results

### 3.1 Subjects information

We initially invited 399 students aged 9 ∼ 18 from Suoga Primary and Middle School (195 males and 204 females) from Liuzhi, Liupanshui, Guizhou, China, an area with endemic coal-burn-pollution-associated dental fluorosis, to participate in this study. However, only 46 individuals (30 males, 16 females, including 28 Miao ethnicity students) met the inclusion criteria, and both students and their parents voluntarily participated in this study (Table 1 and Supplementary Table 1). Minority Miao ethnic group participants were confirmed through inquiries students about being long-term residents of the Miao ethnic group (minority ethnic groups who lived there for at least three generations) and further verification by contacting the parents of participants to review household registration information. Fluorosis patients were diagnosed and classified according to the Dean’s classification system (Supplementary Tables 1, 2). The samples were grouped into Health (H) (Code 0, Dean’s Index) and Dental Fluorosis (F) (Code 3 and Code 4) categories; Healthy Miao (Hm) (Code 0, Dean’s Index) and Dental Fluorosis Miao (Fm) (Code 3 and Code 4). based on the damaging effect of fluoride on teeth as described in the Dean’s Index (Supplementary Table 2). All samples underwent full-length 16S rDNA sequencing.

**TABLE 1:** Characteristics of the study subjects.

Group	Dental fluorosis (*n* = 23)	Healthy (*n* = 23)
Dean’s index	Code 3/Code 4	Code 0
Age*	14.52 (1.68)	13.04 (1.85)
Female^#^	8	8
Male^#^	15	15
Miao^#^	15	13
Other ethnic groups^#^	8	10

*Data are shown as mean (SD).

^#^Data are shown as people’s number.

### 3.2 Summary of sequencing data

We employed the PacBio Sequel II platform to sequence the full-length 16S rDNA (16S ribosomal DNA) from the dental plaques of 46 subjects, generating a total of 895,702 reads. Following removing adapter sequences and quality control procedures, 660,389 high-quality reads were obtained, with an average of 14,356 reads per sample. The average sequence length was 1,460 bp, ranging from 889 to 2,036 bp. The QIIME 2 (2019.4) DADA2 software was utilized to generate a total of 12,007 amplicon sequence variants (ASVs), with an average of 261 ASVs per sample. The rarefaction curves plateaued, indicating sufficient sequencing depth to reflect the diversity present in the samples (Supplementary Figure 2).

### 3.3 Comparison of bacterial community structure and bacterial composition between the healthy and fluorosis groups

A total of 12 phyla, 27 classes, 42 orders, 60 families, 124 genera, and 403 species were identified from all samples. Among these, the top 10 phyla by average abundance were *Proteobacteria* (37.824%), *Firmicutes* (31.304%), *Bacteroidetes* (18.165%), *Fusobacteria* (7.798%), *Actinobacteria* (3.682%), *Saccharibacteria_(TM7)* (0.635%), *Absconditabacteria_(SR1)* (0.236%), *Gracilibacteria_(GN02)* (0.196%), *Spirochaetes* (0.144%), and *Synergistetes* (0.008%), accounting for 99.992% of the total sequences. The top 10 genera by average abundance were *Streptococcus* (21.131%), *Neisseria* (15.950%), *Aggregatibacter* (7.794%), *Haemophilus* (6.832%), *Capnocytophaga* (5.983%), *Fusobacterium* (4.591%), *Prevotella* (4.277%), *Alloprevotella* (3.852%), *Porphyromonas* (3.256%), and *Veillonella* (3.233%), accounting for 76.899% of the total sequences. The top 10 species by average abundance were *Haemophilus parainfluenzae* (5.135%), *Streptococcus sanguinis* (3.960%), and *Streptococcus oralis subsp._dentisani_clade_058* (3.663%), *Veillonella parvula* (2.547), *Alloprevotella Sp._HMT_473* (2.425%), *Prevotella Intermedia* (1.987%), *Lautropia mirabilis* (1.924%), *Fusobacterium periodonticum* (1.753%), *Abiotrophia defectiva* (1.732%), and *Aggregatibacter Aphrophilus* (1.442%), accounting for 26.568% of the total sequences.

**FIGURE 1:**
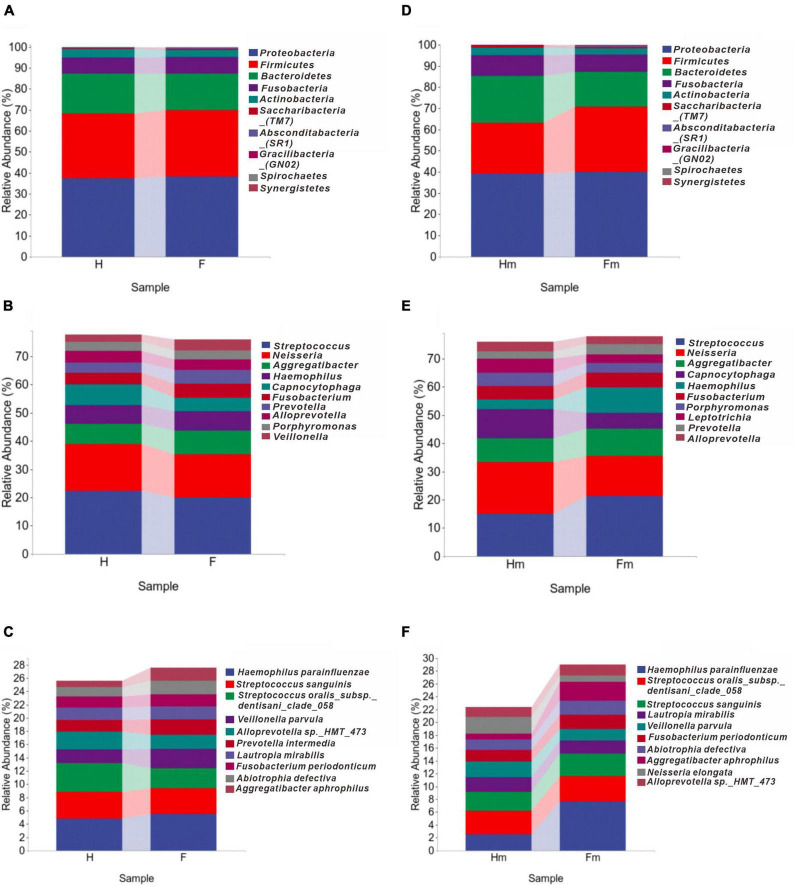
The distributions of major bacterial compositions at different taxonomic levels. **(A–C)** Display histograms of the relative abundance of bacteria in healthy and dental fluorosis groups at the phylum, genus, and species levels. Similarly, **(D–F)** show the bacterial abundances of the healthy and dental fluorosis Miao groups at the same taxonomic levels. The top ten main taxa in relative abundance are shown in each panel.

**FIGURE 2:**
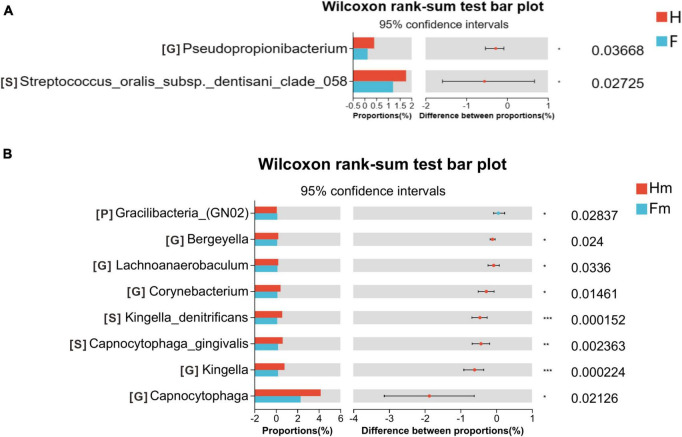
Wilcoxon rank-sum test bar plot on the phylum, genus, and species levels. **(A)** Results of the H and F groups. **(B)** Results of the Hm and Fm groups. The top 10 species at the phylum level and the top 30 species at the genus and species levels were selected for the difference test. The significance of these differences was denoted as follows: *P < 0.05, **P < 0.01, and ***P < 0.001.

To compare the microbial composition differences between the healthy group (H) and the fluorosis patients (F), as well as the Miao healthy group (Hm) and the Miao fluorosis group (Fm), we plotted stacked histograms of the top 10 species’ relative abundance at the phylum, genus, and species levels (Figure 1). At the genus level, *Pseudopropionibacterium* and at the species level, *Streptococcus oralis_subsp.dentisani_clade_058* were more abundant in group H than in group F (*P* < 0.05) (Figure 2A). In contrast, the Hm group had higher levels of *Capnocytophaga*, *Kingella*, *Corynebacterium*, *Bergeyella*, and *Lachnoanaerobaculum* at the genus level compared to the Fm group (*P* < 0.05) (Figure 2B). Furthermore, *Kingella denitrificans* and *Capnocytophaga gingivalis* were more abundant in the Hm group at the species level (*P* < 0.05) (Figure 2B). It is noteworthy that *Gracilibacteria_(GN02)* had a higher relative abundance at the phylum level in the Fm group (*P* < 0.05) (Figure 2B).

**FIGURE 3:**
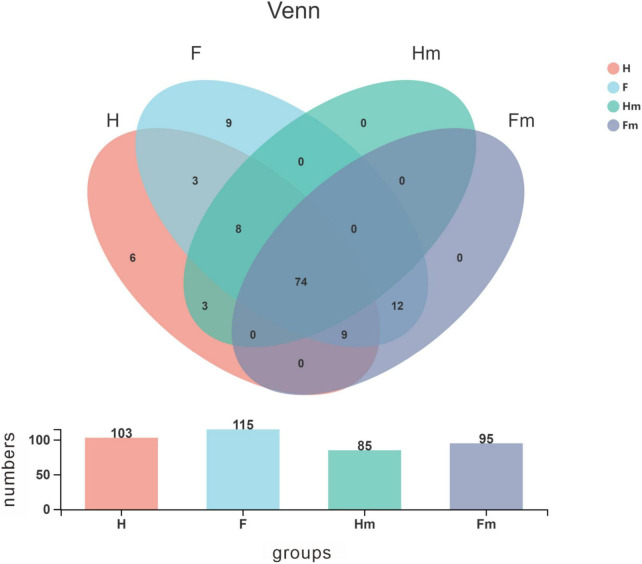
Venn diagram illustrating the distribution of bacterial genera among the four groups. Different colors represent distinct groups, and the bar chart depicts the number of species within each group. The horizontal axis corresponds to the various groups, while the vertical axis represents the species count.

A Venn diagram was utilized to identify the core microorganisms among different groups at the genus level. A total of 103, 115, 85, and 95 genera were identified in the H, F, Hm, and Fm groups, respectively. Notably, 74 genera coexisted in all four groups, representing the core microbiome and accounting for 59.7% of all genera. The remaining 50 non-shared genera were defined as variable microorganisms, including 6 genera exclusive to the H group and 9 genera unique to the F group. All genera in the Hm and Fm groups were encompassed within the H and F groups. There were 94 shared genera in the H and F groups, with 9 genera unique to the H group and 21 genera exclusive to the F group. Additionally, we found that 12 genera were present in both the F and Fm groups but absent from the Hm and H groups. These 12 genera included *Propionibacteriaceae_[G-2]*, *Olsenella, Bacteroidetes_[G-4]*, *unclassified_Bacteroidetes*, *Oribacterium*, *Eggerthia*, *Erysipelotrichaceae_[G-1]*, *unclassified_Selenomonadaceae*, *Megasphaera*, *unclassified_Firmicutes*, *unclassified_Neisseriaceae*, and *Saccharibacteria_(TM7)_[G-2]*. Interestingly, three genera, namely *Bacteroides*, *Centipeda*, and *Paracoccus*, were found in both H and Hm groups but not in F and Fm groups (Figure 3).

### 3.4 Alpha diversity and beta diversity analyses

**FIGURE 4:**
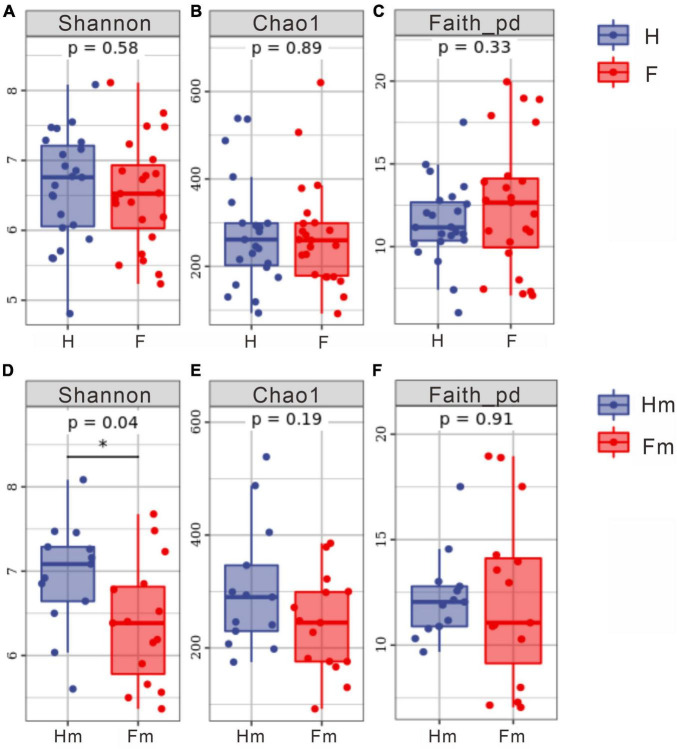
Differences in alpha diversity indices among different groups. **(A–C)** Represent the Shannon index, Chao1 index, and Faith PD index for the healthy group and dental fluorosis group, respectively. **(D–F)** Correspond to the Shannon index, Chao1 index, and Faith PD index for the Miao healthy group and the Miao dental fluorosis group, respectively. The horizontal axis denotes the group labels, while the vertical axis represents the values of the corresponding alpha diversity indices, ranging from the minimum to the maximum, with each box plot illustrating the minimum, first quartile, median, third quartile, and maximum. An asterisk indicates a significant difference (*P* < 0.05).

We analyzed the alpha diversity index of samples from healthy subjects and patients with dental fluorosis. We assessed the diversity (Shannon’s index), richness (Chao1 index), and genetic diversity (Faith’s PD index) of the oral microbiota in different groups (Figure 4). The results revealed no significant difference in the alpha diversity index between the healthy (H) and fluorosis (F) groups. However, comparing the healthy Hm group with the fluorosis Fm group demonstrated a statistically significant difference in the Shannon index (*P* < 0.05, Figure 4D). This finding indicates that oral microbial diversity is more abundant in the healthy Hm group than in the Fm group. Other analyses showed no significant differences in species richness and genetic diversity between the groups. Overall, our results suggest that dental fluorosis is not associated with significant changes in the alpha diversity index, except for a reduction in microbial abundance in the Fm group.

**FIGURE 5:**
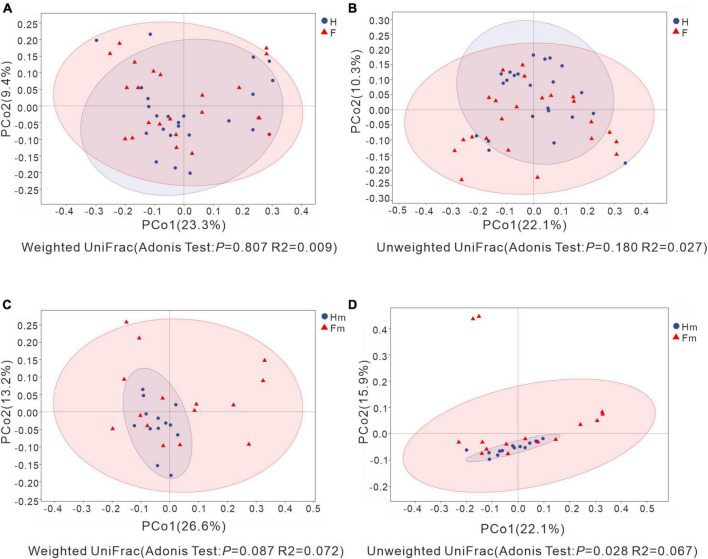
Principal Coordinate Analysis (PCoA) based on Weighted UniFrac distance and Unweighted UniFrac distance. **(A,C)** Represent Weighted UniFrac distance, while **(B,D)** depict Unweighted UniFrac distance. Adonis analysis was utilized to assess the differences in β-diversity indices between groups. The *X*-axis and *Y*-axis explain the largest proportion of variance (expressed as percentages) in bacterial communities, respectively. In each figure, the blue circles represent the healthy group or the healthy Miao group, while the red triangles represent the dental fluorosis group or the dental fluorosis Miao group patient samples. Ovals were calculated and plotted using R language with a confidence level of 0.95.

We analyzed the differences in bacterial communities between patients in the F and H groups using Principal Coordinates Analysis (PCoA). Although some separation was observed between samples from the H and F groups, no significant difference was detected through Adonis analysis (*P* > 0.05) (Figures 5A, B). There was no significant difference between the Hm and Fm groups in the Weighted UniFrac analysis (Figure 5C). In contrast, Unweighted UniFrac-based PCoA analysis revealed that samples from the Hm group were significantly separated from those of the Fm group (Unweighted UniFrac: Adonis test *P* = 0.028, R2 = 0.067) (Figure 5D). This finding suggests that the microbial community structure of dental plaque significantly differed between healthy individuals and patients with dental fluorosis among Miao ethnic individuals.

### 3.5 Microbiology composition heatmap and LEfSe analyses

**FIGURE 6:**
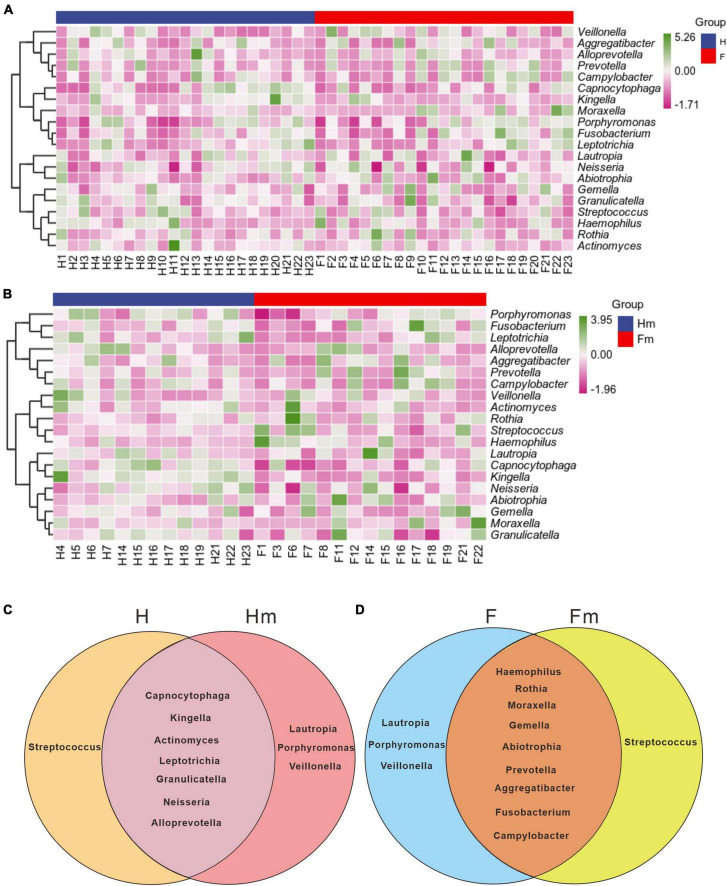
The UPGMA clustering of the species composition heatmap for the top 20 genera at the genus level of panels **(A,B)**, based on the Pearson correlation coefficient matrix of composition data. The blue legend denotes the healthy group or Miao healthy group, while the red legend represents the dental fluorosis group or Miao dental fluorosis group. The dark green and dark purple colors indicate the relative abundance of species, respectively. The Venn diagrams in panels **(C,D)** use different colors to represent the different groups, and the overlapping regions between the two groups can be utilized to identify and enrich the common species of the two groups.

To illustrate the differences in genera composition between the fluorosis and healthy groups, we generated a composition heatmap for the top 20 genera. The top 20 genera in the F and H (Figure 6A) and Fm and Hm (Figure 6B) groups are displayed in Figure 6. We observed that the top 20 genera in mean abundance in the H and F groups were consistent with the genus species composition in the top 20 mean abundance in the Hm and Fm groups. However, the composition of enriched species in each group varied. Next, we employed Venn diagrams to display the unique genera in each group and the common genera in different groups of non-dental and dental fluorosis adolescents. The shared genera of the H and Hm (Figure 6C) and F and Fm (Figure 6D) groups are depicted in the Venn diagram. The results revealed that the H and Hm groups shared seven genera, including *Capnocytophaga*, *Kingella*, *Actinomyces*, *Leptotrichia*, *Granulicatella*, *Neisseria*, and *Alloprevotella*. The F and Fm groups shared nine genera, namely *Haemophilus*, *Rothia*, *Moraxella*, *Gemella*, *Abiotrophia*, *Prevotella*, *Aggregatibacter*, *Fusobacterium*, and *Campylobacter*, which were distinctly different from each other.

**FIGURE 7:**
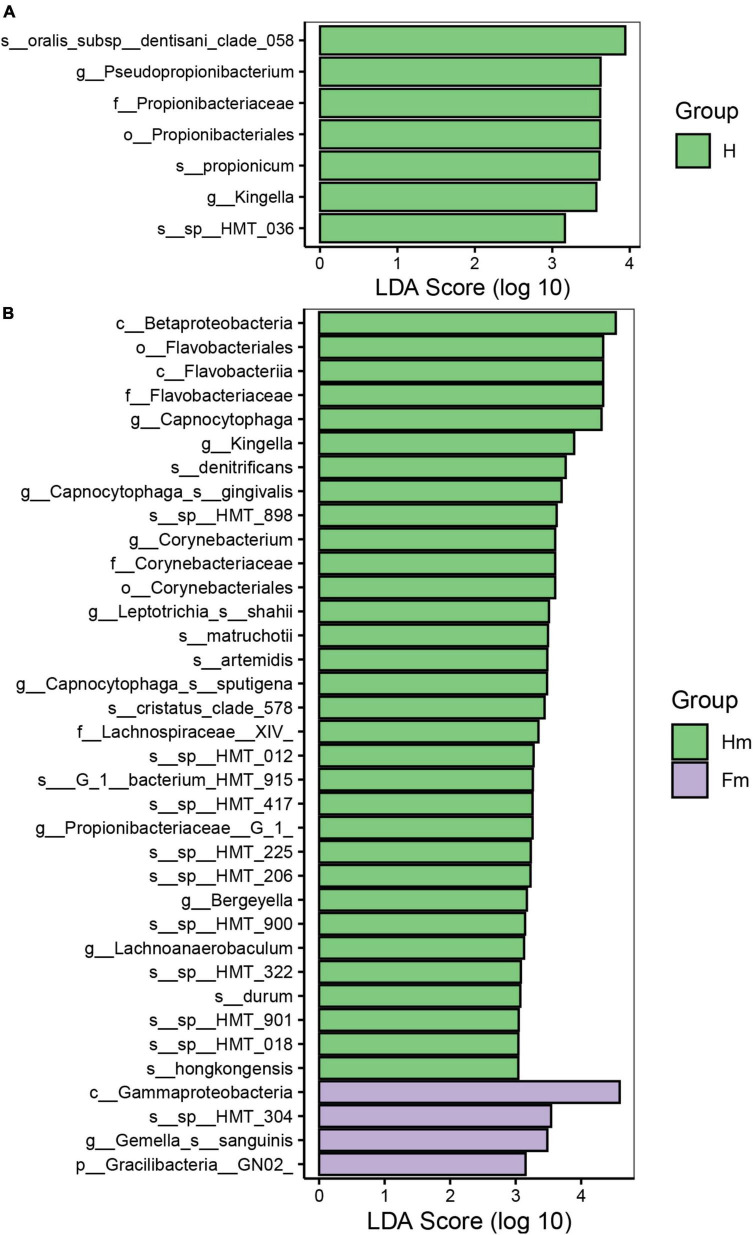
The potential biomarkers defined by LEfSe. **(A,B)** Present the histograms of the Linear Discriminant Analysis (LDA) scores for differentially abundant features among groups. The threshold for discriminative features based on the logarithmic LDA score was set to 3.0.

**FIGURE 8:**
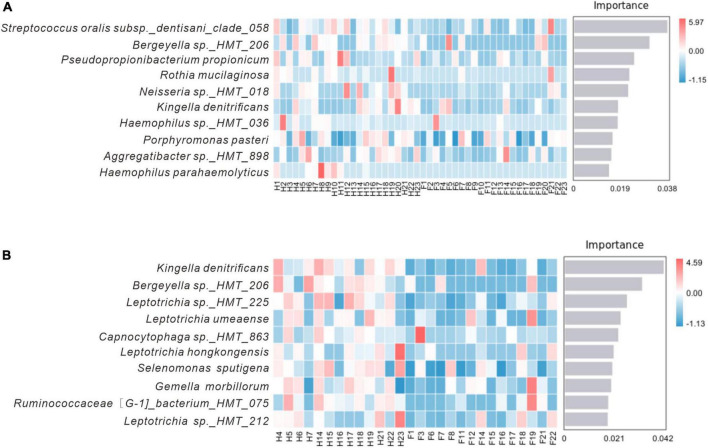
Random forest analysis. **(A)** Represents the H and F group, while **(B)** represents the Hm and Fm group. The horizontal axis of the bar chart displays the importance scores of the species to the classifier model, and the vertical axis shows the names of the taxonomic units at the species level. The heatmap shows the distribution of the abundance of these species across the samples. The species are arranged in decreasing order of importance to the model from top to bottom.

Identifying potential biomarkers between different groups using Linear Discriminant Analysis (LDA), Effect Size Analysis (LEfSe), and Random Forests. Figure 7 shows the histograms of potential biomarkers for different groups (LDA > 3, *P* < 0.05). Notably, we detected significant bacterial differences in samples collected from the Miao ethnic group, with *Gammaproteobacteria* being remarkably enriched in the Fm group at the class level. At the species level, *Prevotella sp_HMT_304* and *Gemella sanguinisen*, and at the phylum level, *Gracilibacteria_(GN02)*, were significantly more abundant in the Fm group. However, it is worth mentioning that we found a total of 32 potential marker species at different levels in the Hm group and 7 potential marker species in the H group. At the same time, *Kingella* (genus) was simultaneously enriched in both healthy groups. Furthermore, the Random Forest analysis revealed that the most important species in the H and F groups was *Streptococcus oralis_subsp._dentisani_clade_058*, while in the Hm and Fm groups, it was *Kingella denitrificans* (Figure 8).

### 3.6 Network analysis and function prediction

**FIGURE 9:**
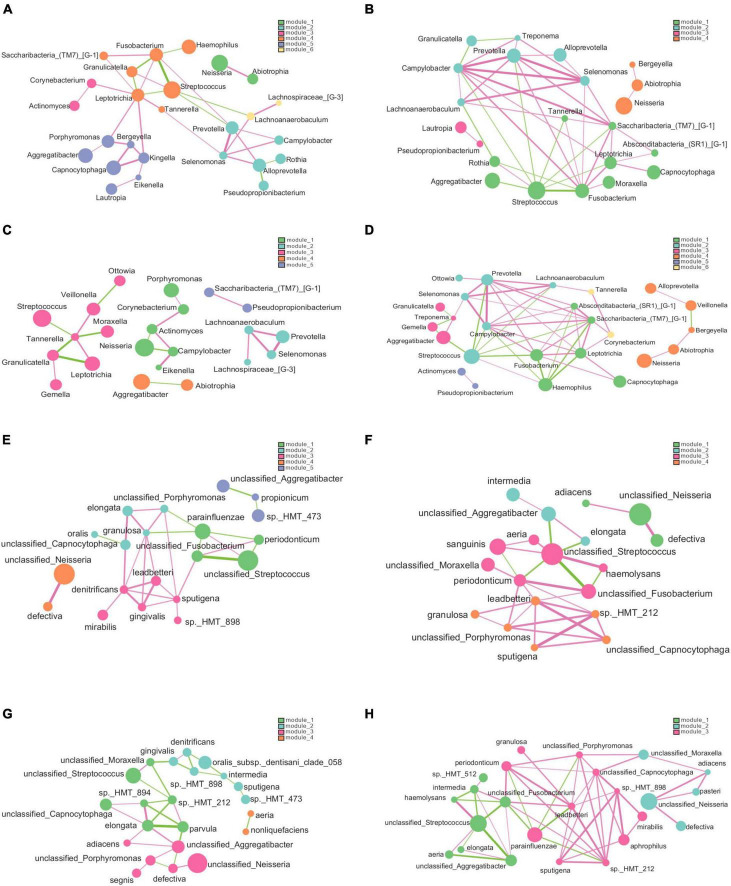
The genus and species-level association networks were constructed using Cytoscape. We set the threshold to 0.6 based on the rank correlation of Spearman, and the abundance was set to 30. **(A–D)** Display the networks constructed by the H, F, Hm, and Fm groups at the genus level, respectively. **(E–H)** Depict the networks constructed by the H, F, Hm, and Fm groups at the species level, where each node represents the genus or species in the sample. The size of the node is proportional to its abundance, and different modules are identified by default in different colors. The edge between nodes indicates a correlation between the two nodes to be connected, and the thickness of the edge reflects the strength of the correlation. Red indicates a positive correlation, while green indicates a negative correlation.

Figure 9 presents the association network we constructed to analyze the relationships among species at the genus and species levels. Our analysis revealed that the structure of the bacterial network in dental plaque of patients with dental fluorosis was more complex, and the relationship between bacteria was more aggregated in the network constructed at either the genus level or species level. Based on the genus-level network, we divided the H and Fm groups into 6 modules; the F group had 4 modules, and the Hm group had 5 modules according to the degree of association between species. In the H group network, 26 genera were related, and *Leptotrichia* was closely associated with 8 other species. It was positively correlated with *Kingella*, a potential core species in the H group. In contrast, *Pseudopropionibacterium*, another potential core species in the H group, was negatively correlated with *Alloprevotella* in the network (Figure 9A). In the F group, 22 genera had interactive relationships, and *Campylobacter* and *Fusobacterium* had complex relationships with 9 species in the network (Figure 9B). The Hm network had 22 genera, and *Tannerella* was positively correlated with *Leptotrichia* and *Veillonella* but negatively correlated with *Streptococcus*, *Moraxella*, and *Granulicatella*. *Corynebacterium*, a potential core species of the Hm group, was positively correlated with Actinomyces and *Porphyromonas*, while *Lachnoanaerobaculum* was positively correlated with *Prevotella*, *Selenomonas*, and *Lachnospiraceae_[G-3]* (Figure 9C). Finally, the Fm network had 25 genera, among which the core species *Prevotella*, *Campylobacter*, and *Saccharibacteria_(TM7)_[G-1]* had complex relationships with the other 9 genera in the network, and the three genera were positively correlated with each other. We also found that *Capnocytophaga*, which was significantly different between the Hm and Fm groups, was positively associated with *Leptotrichia* and *Fusobacterium* in the network (Figure 9D).

The network diagram at the species level revealed 5 modules in the network constructed by the H group, 4 in both the F and Hm groups and 3 in the Fm group. The H group network consisted of 20 species, and the core species *Capnocytophaga sputigena* was positively correlated with 6 species (Figure 9E). In the F group, there were 19 species, and *Fusobacterium periodonticum*, *Capnocytophaga leadbetteri*, and *unclassified_Streptococcus* had complex relationships with the other 7 species in the network (Figure 9F). The Hm group network comprised of 22 species, among which *Neisseria elongata* had complex correlations with 6 species, and *Streptococcus oralis_subsp._dentisani_clade_058* was negatively correlated with *Prevotella intermedia* and *Kingella denitrificans* (Figure 9G). Notably, within the Hm group, the potential biomarkers *Capnocytophaga gingivalis* and *Kingella denitrificans* exhibited a positive correlation. Finally, the Fm network diagram contained 24 species, and the core species *Capnocytophaga leadbetteri* had a complex relationship with nine other species (Figure 9H).

We utilized the PICRUSt2 approach based on the KEGG database to predict changes in microbial function that could be associated with changes in ASV abundance detected by 16S sequencing. This method has proven effective in predicting biological genomes in samples and could offer new insights into the potential functions of oral microbiota. Based on the obtained functional unit abundance table, we created a heat map of each group’s top 30 functional units (Supplementary Figure 3). The clustering of functional units in the heatmap demonstrates a distinct trend between the different groups of samples, indicating the possibility of functional differences between the two groups.

**FIGURE 10:**
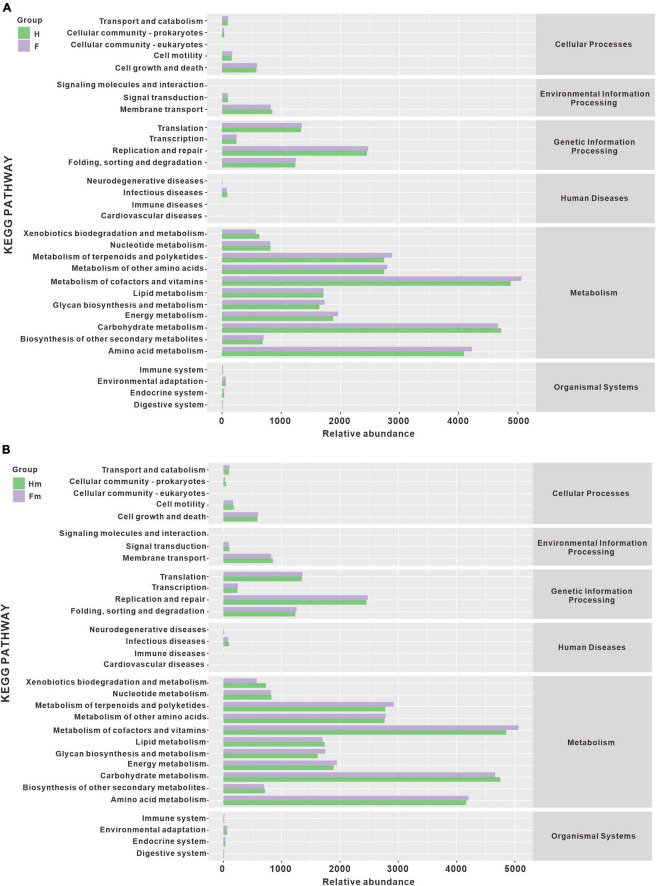
The histogram of metabolic pathway abundance statistics. **(A)** Represents the metabolic pathway statistics of the H and F groups, while **(B)** represents the metabolic pathway statistics of the Hm and Fm groups.

Additionally, we performed statistical analysis on the relative abundance of KEGG pathways and observed similar potential functions for the four bacterial groups (Figure 10). The majority of the gene families were related to the Metabolism of cofactors and vitamins (14.544%), Carbohydrate metabolism (13.732%), Amino acid metabolism (12.168%), Metabolism of terpenoids and polyketides (8.219%), Metabolism of other amino acids (8.095%), Replication and repair (7.203%), Energy metabolism (5.615%), Lipid metabolism (5.016%), Glycan biosynthesis and metabolism (4.947%), and Translation (3.933%). However, we did not observe significant differences in metabolic pathways among the groups (Supplementary Figure 4).

## 4 Discussion

Endemic fluorosis is a chronic systemic disease that poses a serious human risk. Excessive fluoride in water, soil, and food ingested through the digestive tract or respiratory tract can cause enamel cells to develop abnormally, which not only results in the loss of enamel morphology on the surface of our teeth but also affects the function of the teeth. In the present study, we employed third-generation sequencing technology to reveal significant variations in bacterial microbiomes between fluorosis and non-fluorosis adolescents. Compared with second-generation high-throughput sequencing, the full sequence of the 16s RNA gene can reduce the false positive results of bacterial classification caused by sequence similarity. It may have more advantages in the analysis of bacterial diversity (Jeong et al., 2021). Based on the LEfSe analysis, the potential core biomarkers in the oral of Fm fluorosis patients were identified. Network analysis revealed that the microbiota in the fluorosis group exhibited more complex interactions with each other than the healthy group. Our work showed significant differences in the bacterial microbiome between dental plaque samples from healthy Miao adolescents and fluorosis patients. This holds significant implications for devising preventive and therapeutic measures against dental fluorosis.

Although the species composition of the oral microbiota was similar between healthy individuals and those with dental fluorosis, the relative abundance of certain species was different. At the genus level, *Pseudopropionibacterium* and at the species level, *Streptococcus oralis_subsp.dentisani_clade_058* had lower abundance in the F group than in the H group (*P* < 0.05). Our data showed that *Haemophilus*, *Rothia*, *Moraxella*, *Gemella*, *Abiotrophia*, *Prevotella*, *Aggregatibacter*, *Fusobacterium*, and *Campylobacter* were enriched in the F group. At the same time, *Aggregatibacter*, *Haemophilus*, and *Fusobacterium* ranked among the top ten in abundance. *Fusobacterium*, *Prevotella*, and *Aggregatibacter* are anaerobic gram-negative bacteria commonly found in the oral cavity and associated with the development of periodontal disease. *Fusobacterium spp* can ferment carbohydrates and proteins to produce butyrate, while *Prevotella spp* ferments carbohydrates to produce acetate. This may create a favorable environment for the growth of eosinophilic microbes.

Notably, *Streptococcus oralis_subsp._dentisani_clade_058* and *Kingella denitrificans* were the most important marker species for differences between healthy and fluorosis patients. *Streptococcus oralis_subsp._dentisani_clade_058* belongs to a subspecies under *Streptococcus oralis*, first isolated from caries-free dental plaque (Camelo-Castillo et al., 2014). It has been shown to have dual probiotic effects, i.e., antibacterial and antacid, and to inhibit the growth of important oral pathogens such as *S. mutans*, *S. sobrinus*, *Prevotella intermedia*, and *F. nucleatum*. Moreover, it can produce bacteriocins and buffer pH through the arginine solubilization pathway, thus helping to reduce enamel demineralization (López-López et al., 2017; Ferrer et al., 2020). In addition, *Kingella denitrificans*, a Gram-negative bacterium, has been reported to be associated with corneal ulcers, endocarditis, and peritonitis (Kim et al., 2011; Kopyt et al., 2015). In a recent study investigating the subgingival microbiome of patients with rheumatoid arthritis and non-rheumatoid arthritis, *Kingella denitrificans* was found to be more abundant in the non-rheumatoid arthritis group (Lehenaff et al., 2021). We also observed a higher abundance of *Kingella denitrificans* in non-fluorosis patients.

At the species level, *Capnocytophaga gingivalis* was significantly enriched in the Miao healthy group. Studies have reported that *Capnocytophaga* is predominantly present in the oral cavity and is a core microbe in healthy humans (Chen et al., 2018; Xi et al., 2019). Interestingly, the detection rate of *Capnocytophaga* is higher in healthy individuals than in patients with gingivitis or periodontitis (Holdeman et al., 1985; Idate et al., 2022). In the functional prediction analysis, the resulting functional unit heatmap showed a clear clustering pattern for different sample groups, indicating potential functional differences between them. In addition, our analysis revealed the presence of 31 gene families in the plaque samples, with similar composition between the H group and F group, Hm group and Fm group. This information can potentially provide insights into the functional roles of these genes in the context of oral health and disease.

Researching dental fluorosis samples from ethnic minority populations in remote areas of China holds significant importance. In Guizhou Province, Ethnic minority populations often reside in relatively remote and economically underdeveloped areas, where their living environments and lifestyles may differ from other populations. Typical areas of coal-burn endemic fluorosis in Guizhou Province are located in mountainous areas with humid climates. Due to geographical and economic constraints, Miao residents in fluorosis areas still have different degrees of using open stoves with fluorinated coal to cook and dry corn, chili peppers, and pork. These foods have strong fluoride absorption features, and the fluoride content in corn, chili peppers, and rice collected from Miao households is significantly higher than that in non-fluorosis areas (Wei et al., 2006). Coupled with the lack of health awareness among the Miao residents in these endemic areas, the consumption of fluoride-rich foods and poor oral hygiene habits may contribute to the occurrence of dental fluorosis and alter the oral microbiota of affected individuals (Wang et al., 2020). Our research indicates that dental fluorosis may induce alterations in the oral microbiota, potentially influencing the progression of dental fluorosis. Consequently, modulating the composition of oral microbiota stands as a potential approach to mitigate the onset and development of dental fluorosis. Understanding the composition and function of oral microbiota might offer a better comprehension of its relationship with dental fluorosis, thereby providing a scientific basis for the prevention and treatment of this condition.

There are also some limitations in this study. The bacterial enrichment of dental plaque in patients with dental fluorosis is potentially significant for the formation and pathogenicity of dental plaque on the tooth surface. However, further longitudinal studies are needed to determine whether these bacteria promote the clinical manifestation of dental fluorosis and whether they change significantly with the duration of fluoride in the oral environment. Apart from bacteria, other microorganisms, such as fungi, also play a vital role in the oral cavity. For example, *Candida albicans*, commonly found in the oral cavity, can interact with other microorganisms through co-aggregation and co-adhesion, inducing bacterial ecological imbalance and promoting invasive infection (Bertolini et al., 2019; Du et al., 2021; Vadovics et al., 2021). Therefore, further studies are necessary to explore the relationship between other microbiomes and the development of dental fluorosis. In addition, we tried our best to meet the experimental sample size requirements at the experimental design stage. However, due to uncontrollable reasons, a few samples could not be used. In future studies, we will try to design redundant samples to ensure the quality of data analysis.

## 5 Conclusion

This study investigated the oral microbiota a healthy group (*H* = 23) and a dental fluorosis group (*F* = 23), and two subgroups of Miao ethnicity: a healthy Miao group (Hm = 13) and a dental fluorosis Miao group (Fm = 15) from China using full-length 16S rRNA sequencing. Our results showed that the composition of oral microbiota was similar between the H and F groups. At the genus level, *Pseudopropionibacterium* and at the species level, *Streptococcus oralis_subsp.dentisani_clade_058* were less abundant in group F than in group H (*P* < 0.05). Further analysis revealed that the abundance of *Capnocytophaga gingivalis* and *Kingella denitrificans* was significantly lower in Fm fluorosis patients than in the Hm group (*P* < 0.05). At the species level, *Kingella denitrificans* and *Capnocytophaga gingivalis* were more abundant in the Hm group, while *Prevotella sp_HMT_304* and *Gemella sanguinisen* were more abundant in the Fm group. Network analysis revealed that the bacterial network in dental plaque of patients with dental fluorosis was more complex, with the relationships between bacteria being more aggregated. Functional analysis using PICRUSt2 revealed the potential functions of the oral microbiota. However, no significant differences in metabolic pathways were observed among the groups. Overall, our results suggest that dental fluorosis is associated with changes in the composition of the oral microbiota, with the Hm group exhibiting a more diverse and balanced microbiota than the Fm group. Further studies are needed to investigate the functional implications of these changes and their potential role in the development of dental fluorosis.

## Data availability statement

The datasets presented in this study can be found in online repositories. The names of the repository/repositories and accession number(s) can be found below: https://www.ncbi.nlm.nih.gov/, PRJNA957568.

## Ethics statement

The studies involving humans were approved by the Guizhou Medical University Human Experiment Ethics Committee [No. 2019(18)]. The studies were conducted in accordance with the local legislation and institutional requirements. Written informed consent for participation in this study was provided by the participants’ legal guardians/next of kin.

## Author contributions

SL: Formal analysis, Investigation, Software, Writing – original draft. RS: Data curation, Methodology, Formal analysis, Writing – review and editing. YuH: Resources, Writing – review and editing. TZ: Resources, Writing – review and editing. QsZ: Investigation, Resources, Writing – review and editing. QZ: Investigation, Methodology, Writing – review and editing. FR: Investigation, Methodology, Writing – review and editing. XZ: Investigation, Methodology, Writing – review and editing. YD: Formal Analysis, Resources, Investigation, Methodology, Writing – review and editing. RZ: Formal Analysis, Investigation, Methodology, Writing – review and editing. PiL: Resources, Investigation, Methodology, Writing – review and editing. GC: Data curation, Methodology, Investigation, Writing – review and editing. ZG: Conceptualization, Funding acquisition, Resources, Investigation, Writing – review and editing. PeL: Conceptualization, Funding acquisition, Resources, Methodology, Investigation, Writing – review and editing. YaH: Conceptualization, Resources, Methodology, Investigation, Writing – review and editing. XQ: Conceptualization, Funding acquisition, Resources, Methodology, Investigation, Writing – review and editing. JL: Conceptualization, Formal Analysis, Funding acquisition, Resources, Methodology, Investigation, Writing – review and editing. WH: Conceptualization, Funding acquisition, Resources, Supervision, Writing – original draft, Writing – review and editing.

## Footnotes

1
https://docs.qiime2.org/2019.4/tutorials/

2
http://www.homd.org

3
https://www.ncbi.nlm.nih.gov/sra/

4
https://www.kegg.jp/
