# Supplementary material for: Identifying the oral microbiome of adolescents with and without dental fluorosis based on full-length 16S rRNA gene sequencing
Source: Front Microbiol. 2024 Feb 6;15:1296753. doi: 10.3389/fmicb.2024.1296753 (PMC10876846; doi:10.3389/fmicb.2024.1296753)
Supplement: Supplementary file 1 [file Data_Sheet_1.docx]

Supplementary Material

# Supplementary Data

High-throughput sequencing or other large datasets generated in the present study were deposited in a public data center under the accession number SRP433491 (https://www.ncbi.nlm.nih.gov/Traces/sra_sub/).

# Supplementary Figures and Tables

## Supplementary Tables

**Supplementary Table 1:** Summary of volunteers’ information.

| Group | Sample | Gender | Minority group | Age | Dean's Index | Status |
| --- | --- | --- | --- | --- | --- | --- |
| Healthy | H1 | Female | Chuanqing | 16 | Code 0 | Healthy |
|  | H2 | Female | Chuanqing | 15 | Code 0 | Healthy |
|  | H3 | Female | Han | 14 | Code 0 | Healthy |
|  | H4 | Female | Miao | 13 | Code 0 | Healthy |
|  | H5 | Female | Miao | 12 | Code 0 | Healthy |
|  | H6 | Female | Miao | 13 | Code 0 | Healthy |
|  | H7 | Female | Miao | 13 | Code 0 | Healthy |
|  | H8 | Male | Yi | 14 | Code 0 | Healthy |
|  | H9 | Male | Yi | 15 | Code 0 | Healthy |
|  | H10 | Male | Chuanqing | 17 | Code 0 | Healthy |
|  | H11 | Male | Han | 14 | Code 0 | Healthy |
|  | H12 | Male | Chuanqing | 15 | Code 0 | Healthy |
|  | H13 | Male | Han | 15 | Code 0 | Healthy |
|  | H14 | Male | Miao | 12 | Code 0 | Healthy |
|  | H15 | Male | Miao | 11 | Code 0 | Healthy |
|  | H16 | Male | Miao | 11 | Code 0 | Healthy |
|  | H17 | Male | Miao | 12 | Code 0 | Healthy |
|  | H18 | Male | Miao | 12 | Code 0 | Healthy |
|  | H19 | Male | Miao | 10 | Code 0 | Healthy |
|  | H20 | Male | Hui | 11 | Code 0 | Healthy |
|  | H21 | Male | Miao | 12 | Code 0 | Healthy |
|  | H22 | Male | Miao | 11 | Code 0 | Healthy |
|  | H23 | Female | Miao | 12 | Code 0 | Healthy |
| Dental fluorosis | F1 | Female | Miao | 16 | Code 3 | Fluorosis |
|  | F2 | Male | Han | 15 | Code 3 | Fluorosis |
|  | F3 | Male | Miao | 14 | Code 3 | Fluorosis |
|  | F4 | Female | Hui | 11 | Code 3 | Fluorosis |
|  | F5 | Female | Hui | 13 | Code 3 | Fluorosis |
|  | F6 | Male | Miao | 16 | Code 3 | Fluorosis |
|  | F7 | Male | Miao | 14 | Code 3 | Fluorosis |
|  | F8 | Male | Miao | 17 | Code 3 | Fluorosis |
|  | F9 | Female | Han | 15 | Code 3 | Fluorosis |
|  | F10 | Male | Han | 15 | Code 3 | Fluorosis |
|  | F11 | Male | Miao | 17 | Code 3 | Fluorosis |
|  | F12 | Male | Miao | 15 | Code 3 | Fluorosis |
|  | F13 | Male | Chuanqing | 14 | Code 3 | Fluorosis |
|  | F14 | Female | Miao | 14 | Code 3 | Fluorosis |
|  | F15 | Male | Miao | 15 | Code 3 | Fluorosis |
|  | F16 | Female | Miao | 15 | Code 3 | Fluorosis |
|  | F17 | Male | Miao | 13 | Code 3 | Fluorosis |
|  | F18 | Female | Miao | 13 | Code 3 | Fluorosis |
|  | F19 | Female | Miao | 11 | Code 3 | Fluorosis |
|  | F20 | Male | Yi | 17 | Code 4 | Fluorosis |
|  | F21 | Male | Miao | 16 | Code 4 | Fluorosis |
|  | F22 | Male | Miao | 13 | Code 4 | Fluorosis |
|  | F23 | Male | Hui | 15 | Code 4 | Fluorosis |

46 volunteers who met the inclusion criteria and the specific information of the sample.

### Supplementary Table 2: Diagnostic criteria based on Modified Criteria (Dean, 1942).

| Classification | Code | Diagnostic criteria |
| --- | --- | --- |
| Normal | 0 | The enamel presents the usual translucent semi-vitriform type of structure. The surface is smooth, glossy, and usually of a pale creamy white color. |
| Suspicious | 0.5 | The enamel discloses slight aberrations from the translucency of normal enamel, ranging from a few white flecks to occasional white spots. This classification is utilized in those instances where a definite diagnosis of the mildest form of fluorosis is not warranted and a classification of “normal” is not justified. |
| Very mild | 1 | Small, opaque, paper-white areas scattered irregularly over the tooth but not involving as much as approximately 25% of the tooth surface. Frequently included in this classification are teeth showing no more than about 1 ~ 2 mm of white opacity at the tips of the summits of the cusps of the bicuspids or second molars. |
| Mild | 2 | The white opaque areas in the enamel of the teeth are more extensive but do not involve as much as 50% of the tooth. |
| Moderate | 3 | All enamel surfaces of the teeth are affected and surfaces subject to attrition show marked wear. The brown stain is frequently a disfiguring feature. |
| Severe | 4 | Includes teeth formerly classified as “moderately severe” and “severe”. All enamel surfaces are affected, and hypoplasia is so marked that the general form of the tooth may be affected. The major diagnostic sign of this classification is the discrete or confluent pitting. Brown stains are widespread, and teeth often present a corroded-like appearance. |

Note: Diagnosis is based on the two or more teeth with the most damage, with some patients classified as a lower class if the classification is controversial.

## Supplementary Figures


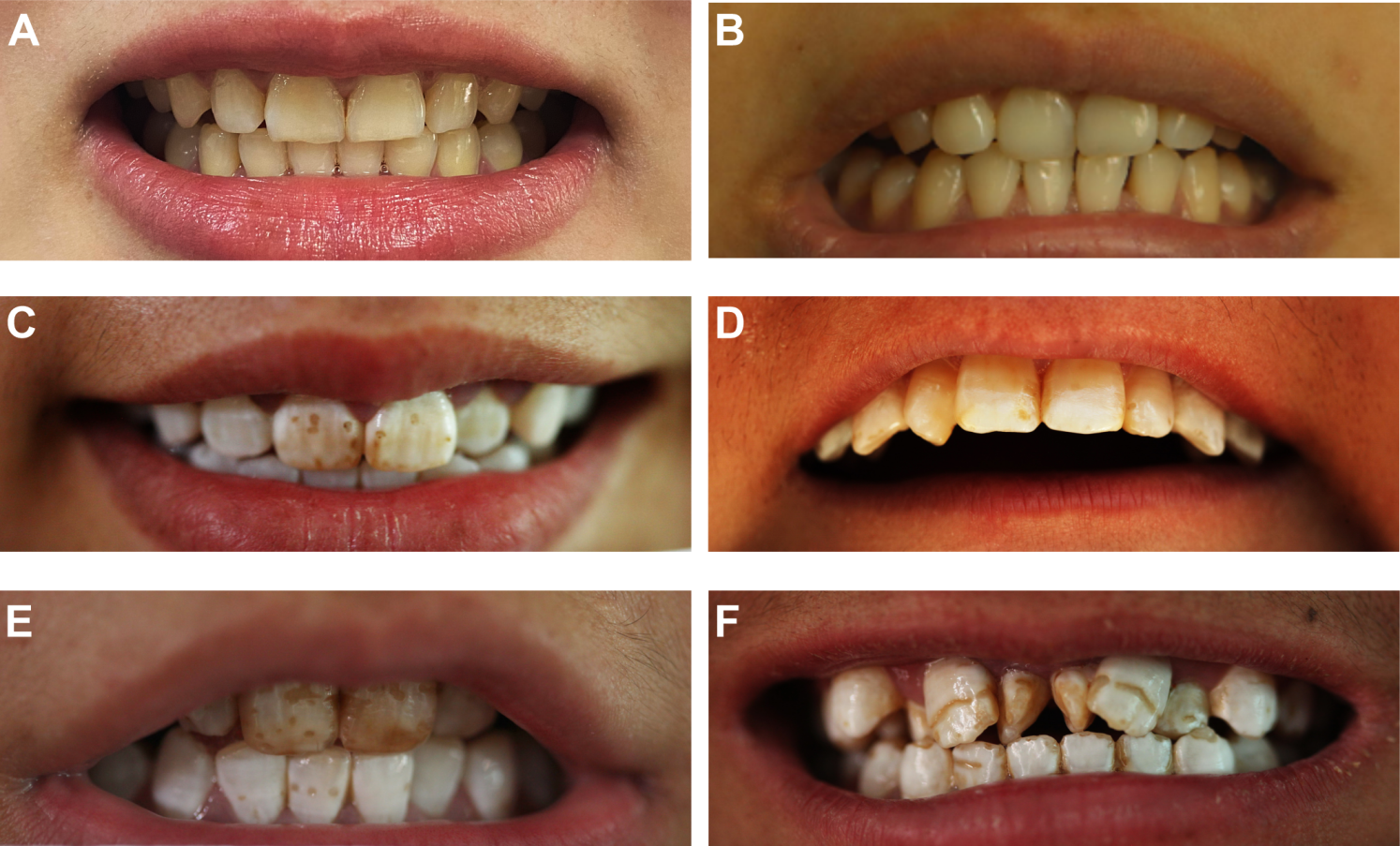


**Supplementary Figure 1.** The typical appearance of teeth in patients with dental fluorosis. (A) and (B) show the appearance of teeth in healthy individuals (code 0); (C) and (D) depict the appearance of teeth in patients with moderate dental fluorosis (code 3); (E) and (F) represent the appearance of teeth in patients with severe dental fluorosis (code 4).


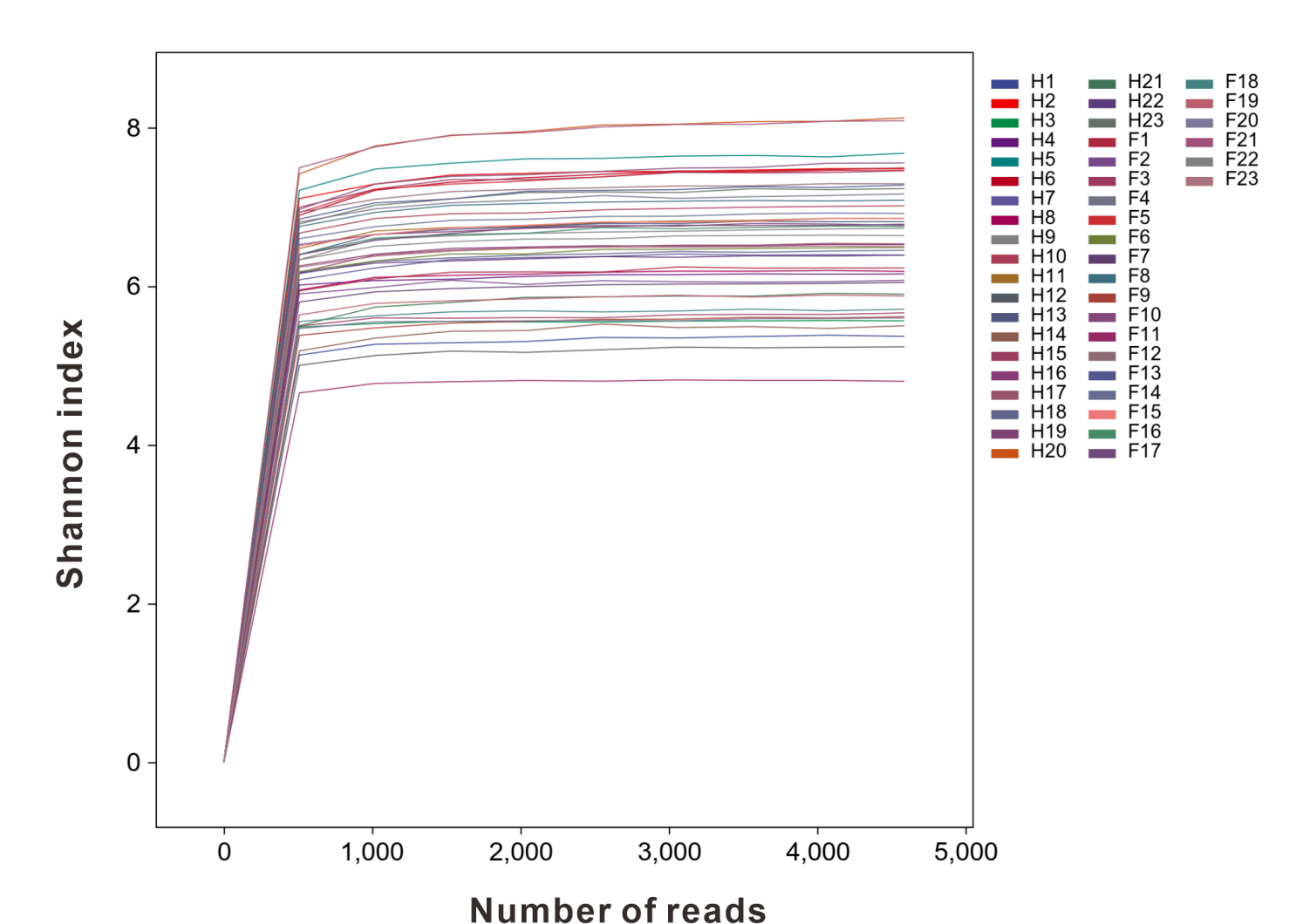


**Supplementary Figure 2.** The rarefaction curve plotted with the Shannon index, which assesses the diversity of bacterial communities within the samples. The depth of sequencing reads is depicted along the horizontal axis, while the Shannon index is displayed along the vertical axis. The rarefaction curve has plateaued, indicating that the sequencing depth was sufficient to capture the majority of the bacterial diversity present in the samples.


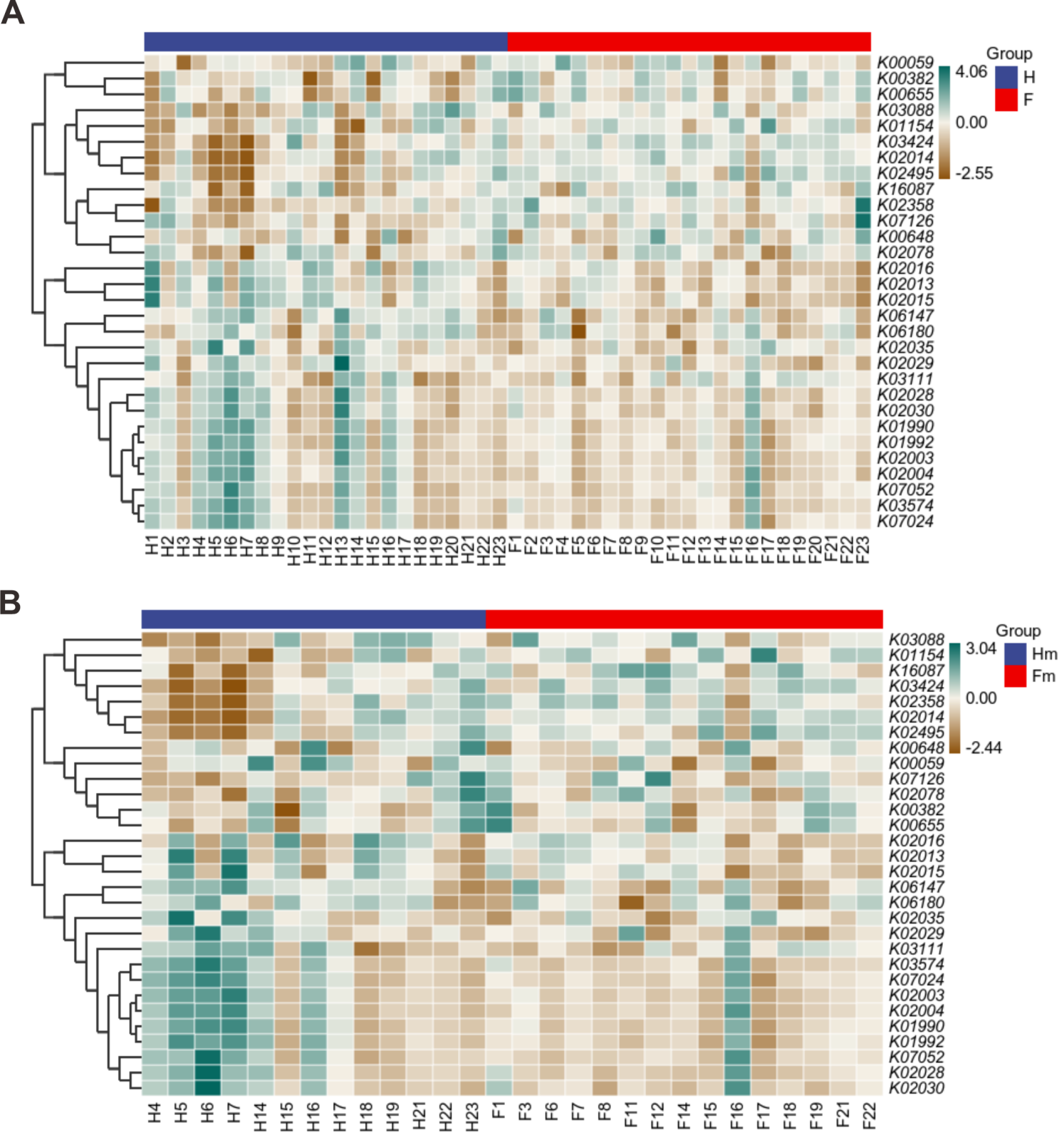


**Supplementary Figure 3.** The heatmap depicting the top 30 functional units in terms of abundance. The data has been normalized using a Z-score, and clustered by row. Figure A shows the distribution of each functional unit in the samples of groups H and F, while Figure B presents the distribution of each functional unit in the samples of groups Hm and Fm.


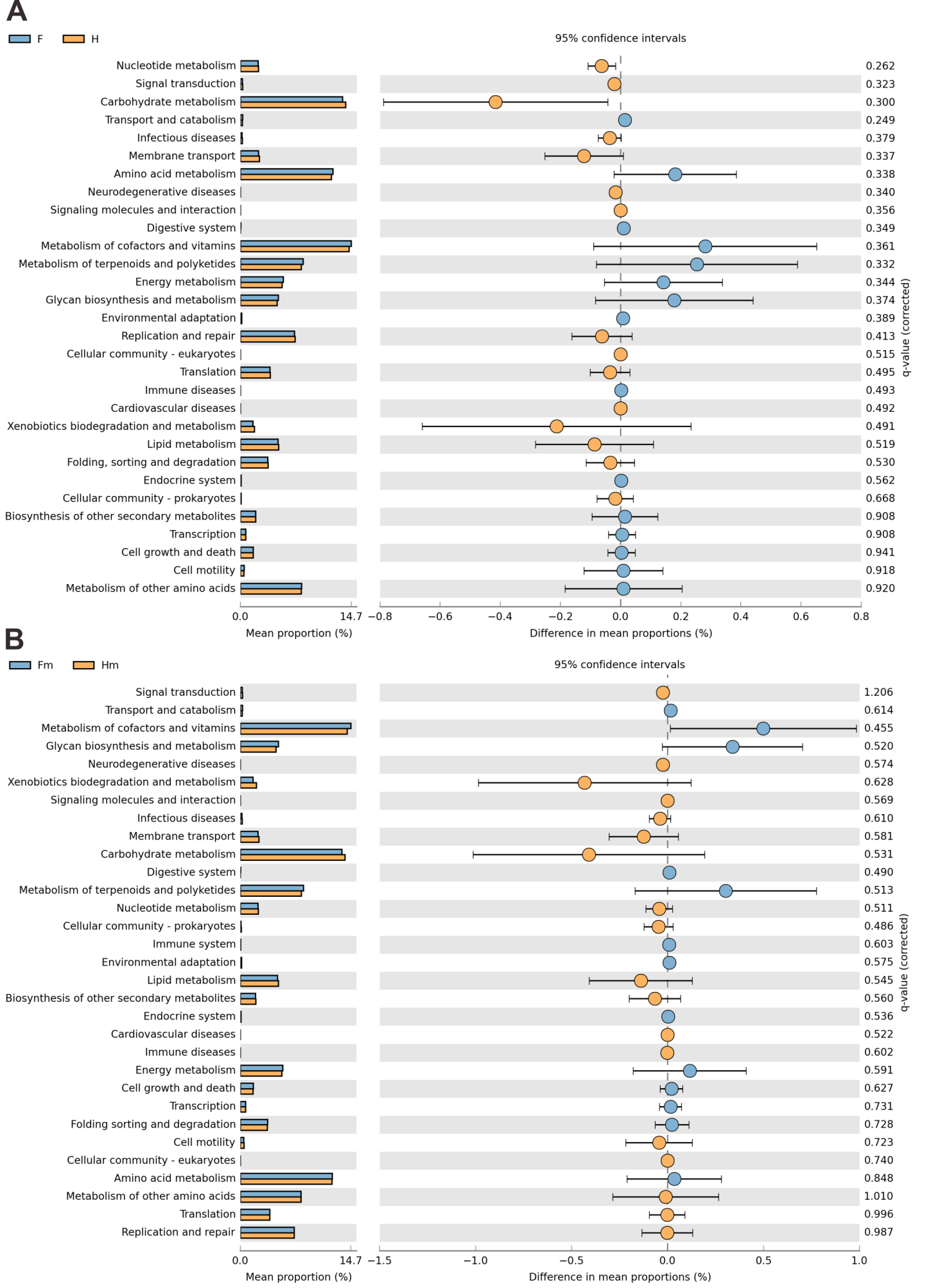


**Supplementary Figure 4.** The results of Welch’s t-test on Metabolic pathways. Panel A displays the test results of the H and F groups, while Panel B shows the metabolic pathway test results of the Hm and Fm groups. We controlled the false positive rate using the Benjamini-Hochberg false discovery rate (FDR) method.
